# Expression of a novel NaD1 recombinant antimicrobial peptide enhances antifungal and insecticidal activities

**DOI:** 10.1038/s41598-024-73710-3

**Published:** 2024-10-05

**Authors:** Sara Royan, Reza Shirzadian-Khorramabad, Arash Zibaee, Mohammad B. Bagherieh-Najjar, Farhad Nazarian-Firouzabadi

**Affiliations:** 1 Department of Agricultural Biotechnology, Faculty of Agricultural Sciences, University of Guilan, Rasht, Iran; 2 Department of Agricultural Biotechnology, Faculty of Agricultural Sciences, University of Guilan, Rasht, Iran; 3 Department of Plant Protection, Faculty of Agricultural Sciences, University of Guilan, Rasht, Iran; 4 Department of Biology, Faculty of Science, Golestan University, Gorgan, Iran; 5 Production Engineering and Plant Genetic Department, Faculty of Agriculture, Lorestan University, Khorramabad, Iran

**Keywords:** Antifungal effect, Antimicrobial peptide, CBD, Defensin, Insecticidal effect, and NaD1, Biotechnology, Molecular biology, Plant sciences

## Abstract

This study aimed to increase the antifungal and insecticidal activities of NaD1, as an antimicrobial peptides (AMP), by improving its interaction with the fungal cell wall and chitin monomeric units in insect midguts. Hence, the chitin-binding domains (CBDs) of wheat germ agglutinin protein (WGA) were fused to either N- or C-terminus of NaD1 generating transgenic *Nicotiana tabacum* hairy roots (HRs). Molecular assessments confirmed the integration of *NaD1* transgenes, their transcription and production of recombinant peptides in the HR lines. Total protein of *(CBD)*_4_*-NaD1* and *NaD1-(CBD)*_4_ transgenic lines inhibited the growth of *Pyricularia oryzae* mycelium, suggesting that fusion of CBD to NaD1 can increase NaD1 half-life, leading to higher affinity toward cell wall chitin. Furthermore, feeding the third-instar larvae of *Chilo suppressalis* with both (CBD)_4_-NaD1 and NaD1-(CBD)_4_ extracts exhibited a higher mortality rate. Both NaD1-CBDs caused a significant decrease in trypsin (TRY) and chymotrypsin (CTR) activities in the larvae, while enhancing the activity of antioxidant enzymes CAT, POD, APX, and SOD. Therefore, feeding the larvae by total extract of NaD1-(CBD)_4_ and (CBD)_4_-NaD1 HR lines probably increased affinity to midgut chitin in *C. suppressalis*, enhancing insecticidal activities. Overall, the results indicate that recombinant peptides are effective in enhancing fungal and insect resistance.

**Supplementary Information:**

The online version contains supplementary material available at 10.1038/s41598-024-73710-3.

## Introduction

Plant diseases caused by viruses, bacteria, fungi, and pests result in a reduction in the quality and quantity of agricultural products^1^. While traditional methods of controlling plant pathogens often involve the use of pesticides, this approach can inadvertently contribute to the development of resistant pathogens and have adverse long-term impacts on human health and the environment^2^. As a result, there is a growing demand for ecologically sustainable crop protection strategies, promoting the exploration of alternative methods, such as non-chemical controls^1,3^.

Natural antimicrobial peptides (AMPs) possess diverse abilities to control infections and can serve as effective alternatives to combating various pathogens^4^. As a result, they have gained popularity for use^5^. AMPs have been found to exhibit broad-spectrum antimicrobial activity, making them excellent candidates for the development of new antimicrobial agents. Moreover, the unique mechanism of action of AMPs, which involves damaging pathogen membranes and targeting intracellular components, makes them less susceptible to resistance development, compared to conventional antibiotics^6,7^. Currently, the applications of AMPs in various fields, including medicine, agriculture, and food preservation are being explored^2,4,5,8^. The use of AMPs can enhance crop resistance to biotic and abiotic stresses, providing significant benefits in plant breeding^9^. This advantage not only improves crop yield and quality but also reduces the reliance on harmful pesticides. Additionally, the use of AMPs can promote more sustainable agricultural practices and contribute to global food security^2,4,5^.

Production of AMPs in bacteria poses challenges due to their bactericidal properties. Plants offer a cost-effective, stable, and safe alternative system for expressing recombinant products. The resulting products are biologically active and retain their natural properties^10,11^. However, the production of recombinant proteins, such as AMPs in transgenic crops before analyzing their activity is both costly and time-consuming. Furthermore, the success of such an approach is uncertain, making it crucial to synthesize proteins and investigate the effects of recombinant proteins in vivo. Hairy roots (HRs) provide a viable solution for producing recombinant peptides and proteins. HRs enable the evaluation of various aspects of gene expression due to their high phenotypic and biochemical stability, rapid biomass production at commercial levels, and long-term sustainability of gene expression^11–13^.

Several strategies have been proposed to enhance the accumulation and stability of peptides in vivo. However, the most significant factor affecting the stability of recombinant peptides is the signal sequence derived from the host or closely related organisms. This sequence is responsible for transferring the product to the extracellular space^12^. Additionally, the secretion of AMPs may enhance the interaction between the peptide and plant pathogenic microorganisms, thereby impeding tissue colonization^12^. To overcome proteolytic degradation, AMPs can be expressed as fusions with other proteins and peptides^10,11^. Various synthetic AMPs have been developed to improve stability and specificity, as well as to boost plants’ resistance to pathogens^14^. Recently, various antimicrobial peptides have been combined with each other or with functional proteins to create bifunctional properties and increase antibacterial activity beyond that of individual AMPs. for example, the cecropin A Mag II hybrid antimicrobial peptide demonstrates significantly enhanced antibacterial effectiveness compared to cecropin A or Mag II^15,16^. Recent research shows that engineered peptides can improve resistance to diseases caused by pathogens, leading to the modification of AMPs through fusion with partners^8,10,11,17^. Also, fusing AMPs with chitin-binding protein (Protein module that binds to chitin monomers), which is abundant in the walls of fungal pathogens and midguts of insects, can prevent their proteolytic degradation (the breakdown of proteins or peptides into smaller polypeptides or amino acids). Furthermore, binding to chitin facilitates the access of the peptide to the plasma membrane of the fungi and insect midgut^18–20^.

Defensins are a crucial group of AMPs that play a significant role in various biological functions. They form part of the innate immune system of plants^21^and have the ability to affect and self-incompatibility^22^. For instance, PCP-A1 a specific type of defensin found in the pollen grain coat of Brassica, contributes to self-incompatibility and prevents inbreeding^23,24^. Defensins also assist in alleviating abiotic stress^22^. NeThio1 and NeThio2 defensins in *Nicotiana excelsior*, NpThio1 in *Nicotiana paniculata*, and Tad1 defensin in *Triticum aestivum* enhance salt and cold stress tolerance, respectively^25^. Moreover, defensins function as epigenetic agents and modify the redox state of ascorbic acid^22^. Apart from their antimicrobial properties, they may display insecticidal properties by inhibiting insect digestive enzymes (The midgut of insects contains digestive enzymes, primarily amylases, glycosidases, lipases, and proteases, which possess similar hydrolytic properties)^21^. Defensins consist of small peptides that range from 45 to 54 amino acids and are particularly rich in cysteine. They possess a conserved three-dimensional structure comprising three beta sheets connected to an alpha-helix^26,27^, and they contain four disulfide bonds known as the cysteine-stabilized αβ (CSαβ) motif (peptide segment), which is highly conserved across different species^27^. Defensins are frequently employed to control plant diseases^4^. However, due to their small size and cationic nature, they are susceptible to proteolytic degradation within the cytosol. By safeguarding AMPs, their half-life can be extended, and their efficacy against pathogens can be improved^11^.

One specific defensin, NaD1, has been characterized in the *N. alata*, commonly known as Persian tobacco. It comprises a functional domain of 47 amino acids and is stored in the vacuole^28^. NaD1 is renowned for its potent antifungal properties and its effectiveness against pathogenic fungi^27^. In *N. alata*, the highest level of transcript accumulation occurs in the peripheral cell layers of the premature flower bud^28^. The expression of NaD1 in the outer layers of the flower bud helps safeguard the reproductive structures from potential harm^28,29^. NaD1 has also demonstrated insecticidal properties as it inhibits the digestive enzymes trypsin (TRY) and chymotrypsin (CTR) in *Helicoverpa* larvae and Light-brown apple moth^29,30^. These diverse properties make NaD1 a promising candidate for the development of novel strategies to combat pathogens and pests.

Wheat germ agglutinin (WGA) with four chitin-binding domains (CBD) repeats, belongs to the chitin recognition protein family. It serves a protective function against insects in plants^31^. The CBD domains interact with the chitin component of the insect’s peritrophic membrane, thereby disrupting the cell wall chitin structure^20,31^. Moreover, the CBDs of WGA bind to the N-acetylglucosamine monomer units in the cell wall of chitin-containing pathogenic fungi, leading to swelling, release of cell contents, and lysis of the germ tube cell wall during spore germination^32,33^.

In this study, we fused the coding sequence of NaD1 peptide to four consecutive CBDs of WGA protein and overexpressed the recombinant genes driven by a 35 S promoter (3X) in *N. tabacum* HRs. The objective was to investigate the insecticidal and antifungal effects against two pathogens: *C. suppressalis* Walker larvae and *P. oryzae* fungi. We evaluated the physiological and biochemical antifungal and insecticidal-related characteristics of the recombinant NaD1-(CBD)_4_, (CBD)_4_-NaD1, and NaD1 protein extracts from transgenic tobacco HR lines. The experimental data demonstrated that the recombinant protein extracts from transgenic HR lines containing NaD1-(CBD)_4_ and (CBD)_4_-NaD1 exhibited greater antifungal and insecticidal activity compared to the NaD1 peptide alone. This natural and effective process could potentially be utilized for pest control and fungal management in agricultural settings.

## Results

### Linker length and checking the antimicrobial properties

To choose a flexible linker, three types of linkers were studied, including linkers with 2, 4, or 6 repetitions of EAAAK sequence. Additionally, we explored and modeled two binding orientations to evaluate the CBDs binding capabilities: (CBD)_4_-NaD1 (Binding of CBDs at the N-terminal) and NaD1-(CBD)_4_ (Binding of CBDs at the C-terminal). Proteins that display similar folds to the parent protein, preserving their original function generally receive a score between 0.5 and 1. Based on scores (Table S1), no obvious difference (s) was found among different linkers. Results of this study showed that two repeats of EAAAK were sufficient for strong binding of NaD1 and CBDs^34–37^.

Prior to gene cassettes preparation, we conducted an in silico evaluation of the antimicrobial properties of the recombinant designed cassettes using SVM, Random Forest, Discriminant Analysis, and ANN algorithms from the CAMP database (Tables S2-S5). According to several CAMP database algorithms, the NaD1-(CBD)_4_ and NaD1-(CBD)_4_ cassettes exhibit enhanced antimicrobial properties compared to NaD1, suggesting that the recombinant sequences are suitable antimicrobial candidates for chemical synthesis and further investigation of their activity under in vitro and in vivo conditions.

### Verification of recombinant expression vectors, transgenic and non-transgenic HR lines

**Fig. 1:**
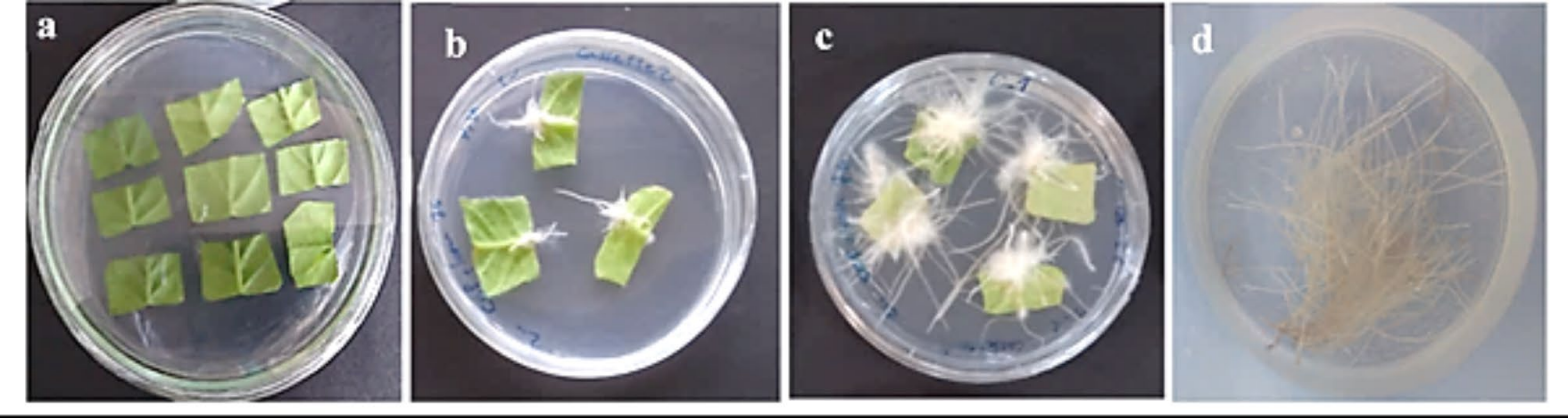
Inoculation of tobacco leaf explants by *Agrobacterium rhizogenes* strain *ACC 15,834* and generation of HRs. (**a**) Co-cultivation of leaf explants and *Agrobacterium rhizogenes* strain *ACC 15,834* containing pGSA1285:*(CBD)*_*4*_*-NaD1*, pGSA1285:*NaD1-(CBD)*_*4*_ and pGSA1285:*NaD1* gene constructs. (**b**) Generation of HRs is shown around the leaf explants on MS medium containing kanamycin, and cefotaxime. (**c**) Cultivation and growth of transgenic HRs on the MS medium containing kanamycin and cefotaxime. (**d**) Growth and development of transgenic HRs in MS liquid medium containing 100 mgL^−1^ of kanamycin and 200 mgL^−1^ of cefotaxime.

**Fig. 2:**
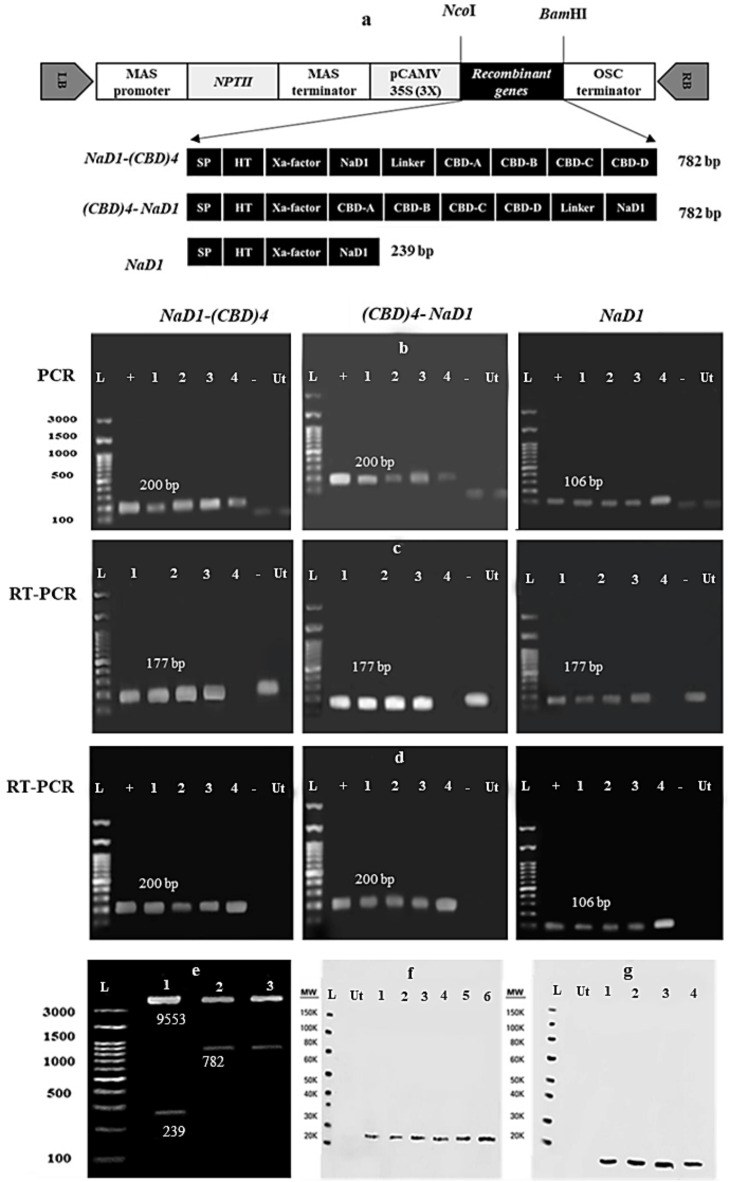
Molecular analysis of HR clones expressing recombinant constructs. (**a**) Recombinant constructs: *MAS*, mannopine synthase; *NPT* II: neomycin phosphotransferase II; CaMV35S(3×) cauliflower mosaic virus 35 S promoter; *OSC*, octopine synthase; SP, vicilin signal peptide; HT, histidine tag; *Xa factor*, TEV tobacco Etch Virus Sequence; *NaD1*, Nicotiana alata Defensin1; *CBD*, chitin-binding domains of wheat germ agglutinin protein; Linker, helix-forming linker (EAAAK)2; RB and LB, right and left borders of Agrobacterium T-DNA region, respectively. (**b**) Amplification of recombinant cassettes, including NaD1-(CBD)_4_, (CBD)_4_-NaD1, and NaD1 using specific primers is shown. Bands of 200 (NaD1-(CBD)^4^ and (CBD)_4_-NaD1) and 106 (NaD1) bp length are shown. The non-transgenic HR (Ut), negative control (-), and a plasmid containing the recombinant peptide cassettes were included as the positive control (+). (**c**) Expression of housekeeping gene *elf1-α* using semi-quantitative RT-PCR. Bands of 177 bp are seen in transgenic and non-transgenic HRs (Ut). (**d**) Semi-quantitative RT-PCR production of 200 bp confirmed expression of *NaD1-(CBD)4* and *(CBD)4-NaD1*, and amplification of 106 bp clarified expression of *NaD1* in the HRs by employing gene-specific primers. Non-transgenic HRs (Ut) or the negative control (-) did not display those bands. (**e**) Double digestion of pGSA1285: *NaD1- (CBD)*_4_, pGSA1285: *(CBD)*_4_*-NaD1*, and pGSA1285: *NaD1* constructs by *Bam*HI and *Nco*I enzymes confirmed the presence of the three genetic cassettes in the expression vector pGSA1285. The observed fragments include 9553 bp, corresponding to the pGSA1285 expression vector, 782 bp, related to the *NaD1- (CBD)*_4_, *(CBD)*_4_*-NaD1* genes, and 239 bp related to the *NaD1* gene. (**f**) The recombinant peptides were identified by utilizing the Anti-His monoclonal antibody sc-8036 (Santa Cruz Biotech). Transgenic HRs (1, 2, and 3) expressed (His)_6_-NaD1-(CBD)_4_; (4, 5, and 6) expressed (His)_6_- (CBD)_4_- NaD1 (~ 27 KDa), (**g**) Transgenic HRs (1, 2, 3, and 4) expressed (His)_6_-NaD1 (~ 9 KDa) peptide. Ut: represents non-transgenic HRs.

Following codon optimization, chemical synthesis, and digestion of pUC57 vectors (pUC57: *NaD1-(CBD)*_*4*_, pUC57: *(CBD)*_*4*_*-NaD1*, and pUC57: *NaD1*) using *Bam*HI and *Nco*I, the coding sequences of three recombinant proteins (NaD1-(CBD)_4_, (CBD)_4_-NaD1, and NaD1) were subcloned into a pGSA1285 vector. To validate the correct integration of *NaD1-(CBD)*_*4*_, *(CBD)*_*4*_*-NaD1*, and *NaD1* in the expression vectors, enzymatic digestion was performed with *Bam*HI and *Nco*I enzymes (Fig. 1e). Additionally, Sanger sequencing was conducted to confirm the precise sequence of the recombinant segments. The engineered gene cassettes (pGSA1285:*(CBD)*_*4*_*-NaD1*, pGSA1285:*NaD1-(CBD)*_*4*_, and pGSA1285:*NaD1*) were introduced into tobacco leaf explants (1 cm^2^) via *Agrobacterium rhizogenes* strain *ACC 15,834*-mediated transformation. Successful transformations were manifested as HRs within approximately 7 days (Fig. 2). Among the 50 inoculated leaf disks for each gene cassette, around 48 produced HRs, indicating a high transformation efficiency of 96% on MS (Murashige and Skoog) medium containing 400 mg l^−1^ cefotaxime and 100 mg l^−1^ kanamycin. Out of the HR clones, 10 clones were randomly selected to confirm the absence of bacterial contamination and to ensure successful transformation. The absence of *A. rhizogenes* contamination in the *NaD1-(CBD)4*, *(CBD)4-NaD1*, and *NaD1* HR clones was confirmed using the *VirD1* primers (Table 1 and Fig. S4). Additionally, the presence of the *rolC* gene was assessed, demonstrating successful transformation (Fig. S5). Finally, four transgenic HRs were randomly selected for further analysis and subsequent experiments. To screen the putative transgenic HR lines, *NaD1-(CBD)*_*4*_, *(CBD)*_*4*_*-NaD1*, and NaD1 specific primers were used, resulting in amplification of a 200 bp product for *NaD1-(CBD)*_*4*_ and *(CBD)*_*4*_*-NaD1*, and a 106 bp product for *NaD1* PCR products (All HRs grown on 100 µg. ml^−1^ kanamycin were transgenic) (Fig. 1b).

**Table 1:** The sequence information of utilized primers in PCR and RT-PCR reactions.

Gene name	Sequences	Annealing temperature (°C)	Expected Size (bp)
*NaD*_1_ *-(CBD)*_4_	Forward:5’- GGTTACTGCGGATTTGGTGC-3’ Reverse:5’- GGCTTGTCAGTGGAACATGC-3’	57	200
*(CBD)*_4_ *-NaD*_1_	Forward:5’- GGATACTGCGGATTTGGTGC-3’ Reverse:5’- GGCTTGTCAGTGGAACATGC-3’	57	200
*NaD*_1_	Forward: 5’-AGGATCTCATCATCACCACCA-3’ Reverse: 5’-AGCTTTTCTACATGGTGGCT-3’	57	106
*rol* C	Forward: 5’-TGCTTCGAGTTATGGGTACA-3’ Reverse: 5’-CTCCTGACATCAAACTCGTC-3’	51	626
*Vir* D_1_	Forward: 5’-ATGTCGCAAGGCAGTAAG-3’ Reverse: 5’-CAAGGAGTCTTTCAGCATG-3’	51	441
*elf1-*α	Forward:5’- TCTTAACCATACCAGCATCACC-3’ Reverse: 5’-TGAACCATCCAGGACAGATTG-3’	54	177

RT-PCR amplification of two fragments of 200 bp and 106 bp, respectively, confirmed the expression of both *NaD1-(CBD)*_*4*_ and *(CBD)*_*4*_*-NaD1*, as well as *NaD1* in the selected transgenic HRs (Fig. 1d). By utilizing the ELISA approach, production and concentration of NaD1-(CBD)_4_, (CBD)_4_-NaD1 and NaD1 recombinant peptides (including [His]_6_ signal) in the transgenic HR lines were approved and identified as follows: 42 µg ml^−1^ g^−1^ fresh weight for NaD1-(CBD)_4_, 180 µg ml^−1^ g^−1^ fresh weight for (CBD)_4_-NaD1 and 700 µg ml^−1^ g^−1^ fresh weight for NaD1, confirming the production of all recombinant peptides in the HR lines. While this value was zero for non-transgenic HR (Supplementary Fig. S2). Moreover, the Western blot analysis showed the presence of (His)6-NaD1-(CBD)_4_ and, (His)6-(CBD)_4_-NaD1 (~ 27 kDa), and (His)6-NaD1 (~ 9 kDa) protein bands in the selected transgenic HR lines (Fig. 1f and g). Subsequently, the transgenic HR lines with the highest recombinant protein synthesis were chosen for further antifungal and insecticidal bioassay experiments.

### Antifungal efficiency of recombinant proteins against Pyricularia oryzae

#### Nad1 expressing recombinant protein demonstrates significant antifungal activity

**Fig. 3:**
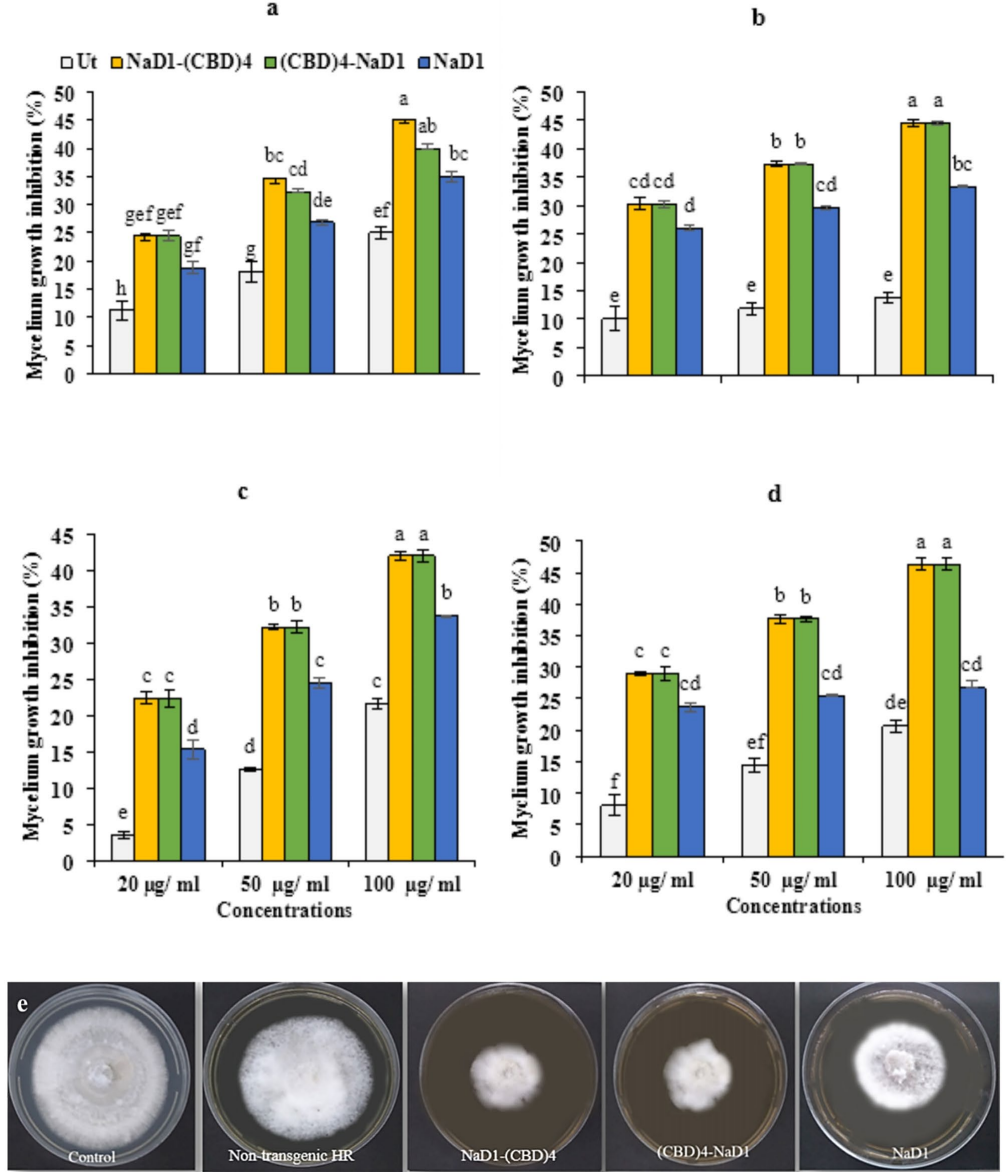
Mycelia growth inhibition was performed by mixing the recombinant proteins with the PDA culture medium. Diagrams depicting the effect of NaD1-(CBD)_4_, (CBD)_4_-NaD1, and NaD1 transgenic and non-transgenic protein extracts on the growth inhibition of *P. oryzae* mycelia 2, 5, 7, and 10 days post treatment (**a**, **b**, **c** and **d** respectively) with 20, 50, and 100 µg/ml of total protein extract from transgenic and non-transgenic HR clones. The percentage of inhibition was determined in comparison to the control group treated with the phosphate buffer. (**e**) The antifungal activity of five protein extracts including three transgenic HRs (NaD1-(CBD)_4_, (CBD)_4_-NaD1, and NaD1), the non-transgenic HR (Ut) (50 µg/ml), and sterilized phosphate buffer (control) was examined against mycelium growth inhibition (*Pyricularia oryzae*) on the seventh day. The results of antifungal activity were subjected to the statistical analysis and the data mean comparison was carried out by the least significant differences (LSD) (*P* < 0.01) in three replicates.

**Fig. 4:**
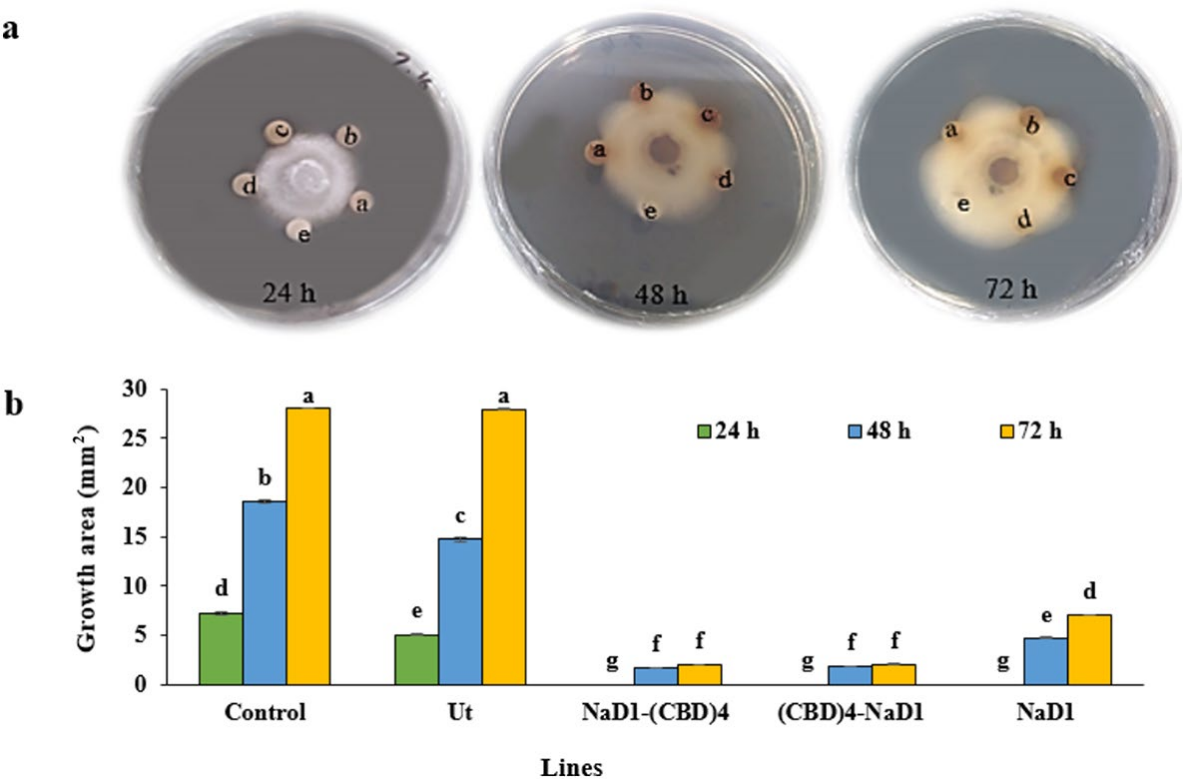
Antifungal activity of the HR protein extract using disc diffusion method against *Pyricularia oryzae*. (**a**) Effect of 50 µg/ml NaD1-(CBD)_4_, (CBD)_4_-NaD1 and NaD1 protein extracts on the growth of *P. oryzae* using the disc diffusion method after 24, 48, and 72 h. (**b**) The effects of 50 µg/ml NaD1-(CBD)_4_, (CBD)_4_-NaD1 and NaD1 protein extracts from HR lines on growth of *P.oryzae* after 24, 48 and 72 h of treatment. a, b, c, d and e include (CBD)_4_-NaD1, NaD1-(CBD)_4_, NaD1, Non-transgenic HR (Ut), and phosphate buffer as a negative control, respectively. Controls include the phosphate buffer as the negative control (Control), and the non-transgenic (Ut) protein extract. Each disc has a surface area of 28.27 mm2. The results of antifungal activity were subjected to statistical analysis and the mean comparison was carried out through the least significant differences (LSD) test (*P* < 0.01) in three replicates utilizing SAS _9.1_ software.

The data analysis of antifungal activity of four protein extracts including the three transgenic HRs (NaD1-(CBD)_4_, (CBD)_4_-NaD1 and NaD1) and the non-transgenic HR at various concentrations of 20, 50, and 100 µg ml^−1^ in the PDA (Potato dextrose agar) medium against the initial formation of mycelium (*Pyricularia oryzae*) on the second, fifth, seventh, and tenth days revealed significant differences (*P* < 0.01) between the four protein extracts, among the three concentrations, and their interactions (Protein extracts * concentrations) (Supplementary Table S7). Comparing the mean data of the three protein extracts in inhibition of *P. oryzae* mycelium growth using least significant differences (LSD) showed a significant difference (*P* < 0.01) among HR clones (Fig. 3). The average growth inhibitory rate of mycelia after inoculation on different days (2, 5, 7, and 10 days) followed a consistent trend (Fig. 3). Throughout all days, the highest mycelium growth inhibition was observed in NaD1-(CBD)_4_, (CBD)_4_-NaD1 extracts, while the lowest growth inhibition was noted in the non-transgenic HR extract particularly evident on the seventh day at a concentration of 50 µg ml^−1^ (Fig. 3e). The maximum inhibition rate was observed at a concentration of 100 µg ml^−1^ (50% growth inhibition), with the lowest at 20 µg ml^−1^ (Fig. 4). This resulted in a 40% higher inhibition rate compared to the non-transgenic HR (Fig. 3). The growth inhibition rate of the NaD1 peptide was approximately 30%, a value approximately 20% higher than that of the non-transgenic extracts and 20% lower than that of NaD1-(CBD)_4_ and (CBD)_4_-NaD1 (Fig. 3). These findings, confirm that incorporating CBDs into the antifungal peptide NaD1 effectively inhibits mycelium growth from the second day of inoculation and could serve as potential fungicide compounds for controlling the growth of *P. oryzae* mycelia.

### Enhanced inhibition of hyphal extension of ***Pyricularia oryzae***

The hyphal extension-inhibition assay was performed to assess the antifungal activity of recombinant proteins. The antifungal activity of protein extracts at a concentration of 50 µg ml^−1^ from the NaD1-(CBD)_4_, (CBD)_4_-NaD1, NaD1 transgenic HRs, non-transgenic HRs, and phosphate buffer (used as a negative control) were evaluated against *P. oryzae* at different time intervals (24, 48, and 72 h) using the disc diffusion method. The analysis of the data revealed a significant difference (*P* < 0.01) among the five extracts, at various time points, as well as in their interaction (protein extracts * times) (Supplementary Table S8). Comparing the mean data of the three transgenic proteins, non-transgenic HR extract, and phosphate buffer in inhibiting hyphal extension showed a significant difference (LSD, *P* < 0.01) (Fig. 4). NaD1-(CBD)_4_, (CBD)_4_-NaD1 and NaD1 protein extracts exhibited the highest inhibition of hyphal extension after 24 h post treatment, with no observable growth on the disc surface containing these extracts (Fig. 4). In contrast, discs containing phosphate buffer and non-transgenic HR extract showed around 25.6% and 17.89% hyphal extension on their surfaces, respectively. Further evaluation of the paper disc surface area showed hyphal extension at rates 65.7% for phosphate buffer, 52.21% for non-transgenic HR, 6% for NaD1-(CBD)_4_, 6.3% for (CBD) _4_-NaD1, and 16% for NaD1 after 48 h. After 72 h, the hyphal extension rate on the surface of the disc containing the extracts were 99.16%, 98.90%, 7.07%, 7.18%, and 24.97%, respectively (Fig. 4b). The protein extracts containing NaD1-(CBD)_4_ and (CBD)_4_-NaD1 showed greater inhibition of hyphal extension in *P. oryzae* compared to the others (Fig. 4). This suggest that, the presence of CBDs in NaD1-(CBD)_4_, (CBD)_4_-NaD1 enhances the fungicidal properties of the recombinant proteins by accumulating more proteins on the chitin walls of *P. oryzae* fungal cells.

### Reduced survival of the third-instar larvae of ***C. suppressalis***

**Fig. 5:**
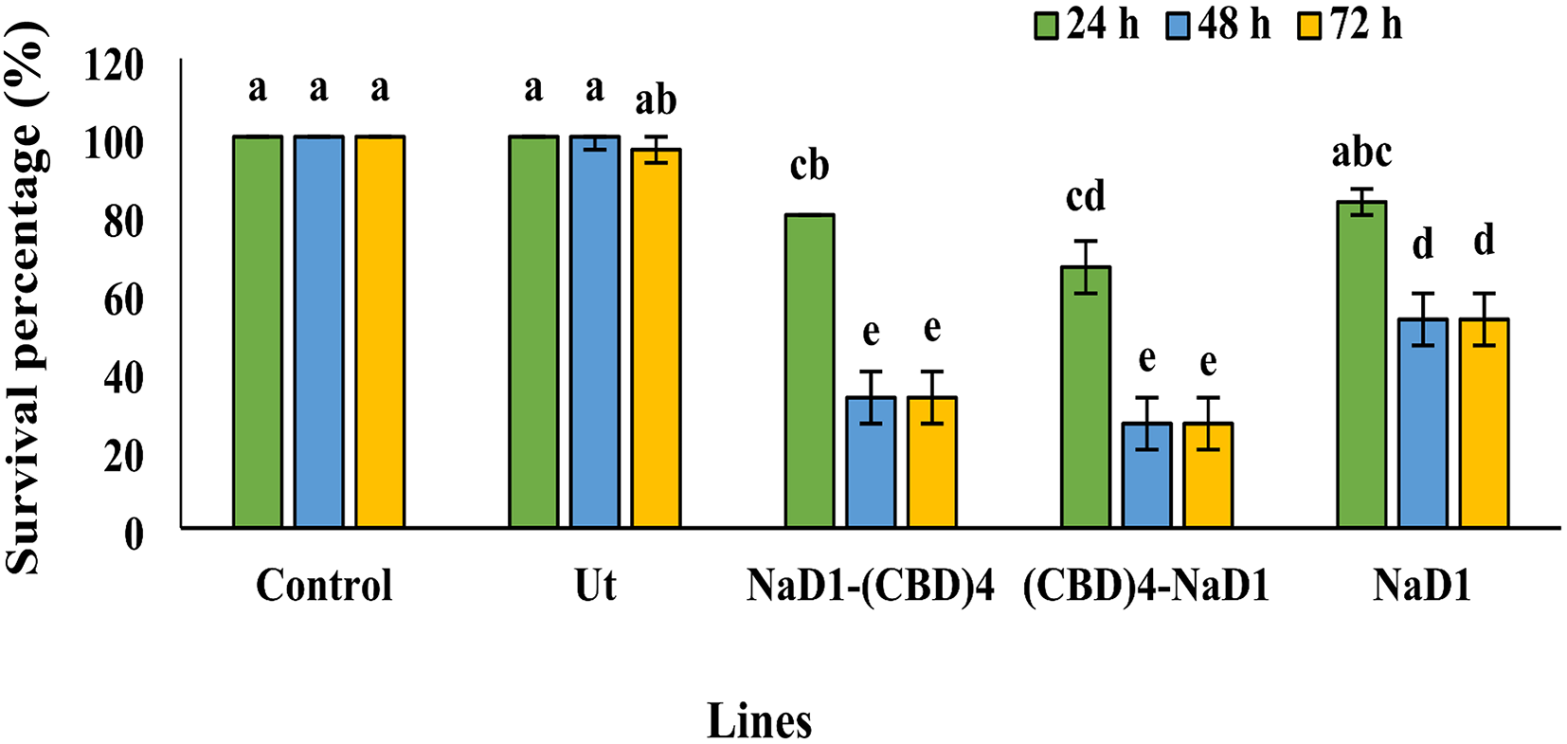
Survival percentage of the third instar larvae of *C. suppressalis* feeding by the HR recombinant protein extracts after 72 h. The impact of 20 µg/ml diverse HR protein extracts, which included transgenic groups (NaD1-(CBD)_4_, (CBD)_4_-NaD1, and NaD1) as well as the HR control comprising phosphate buffer (control) and the non-transgenic HR (Ut) on the survival rate of third instar larvae of *C. suppressalis* was examined. The results of insecticidal activity underwent statistical analysis, and the mean comparisons were conducted using the least significant differences (LSD) test (*P* < 0.01) in three replicates with the assistance of SAS _9.1_ software.

The variance analysis and mean comparison of the survival percentage of third larvae instar of *C. suppressalis* fed by 20 µg/ml protein extracts (NaD1, NaD1-(CBD)_4_, (CBD)_4_ -NaD1, non-transgenic HR extract and phosphate buffer) after 24, 48 and 72 h showed a significant difference (*P* < 0.01) among the protein treatments, and the interaction of protein treatment with time (Supplementary Table S9). Larval survival remained consistently at 100% when fed with rice stalks dipped in phosphate buffer and non-transgenic protein extract at various time points, showing no significant difference (*P* < 0.01) (Fig. 5). However, larvae fed with rice stalks dipped in protein extracts NaD1, NaD1-(CBD)_4_, and (CBD)_4_-NaD1 exhibited significant variation in survival rates (Fig. 5). Notably, NaD1-(CBD)_4_ and (CBD)_4_-NaD1 resulted in the highest mortality (lowest survival percentage) at 48 and 72 h post feeding. While, the survival rate of larvae fed with NaD1 protein extract, did not significantly differ from the others at 24 h. It decreased to 53% after 48 and 72 h, showing a significant difference (*P* < 0.01) compared to NaD1-(CBD)_4_ and (CBD)_4_-NaD1 (33% and 26%, respectively) (Fig. 5). This suggests that the presence of CBD in the recombinant proteins NaD1-(CBD)_4_ and (CBD)_4_-NaD1 enhances their insecticidal properties. Moreover, altering the position of CBD in the recombinant proteins does not affect their insecticidal effectiveness. The insecticidal impact of the extracts appears to be most pronounced 48 h after feeding with the recombinant protein extracts.

### Recombinant protein extracts change activities of CAT, POD, APX, SOD, TRY and CTR enzymes in ***C. suppressalis***

**Fig. 6:**
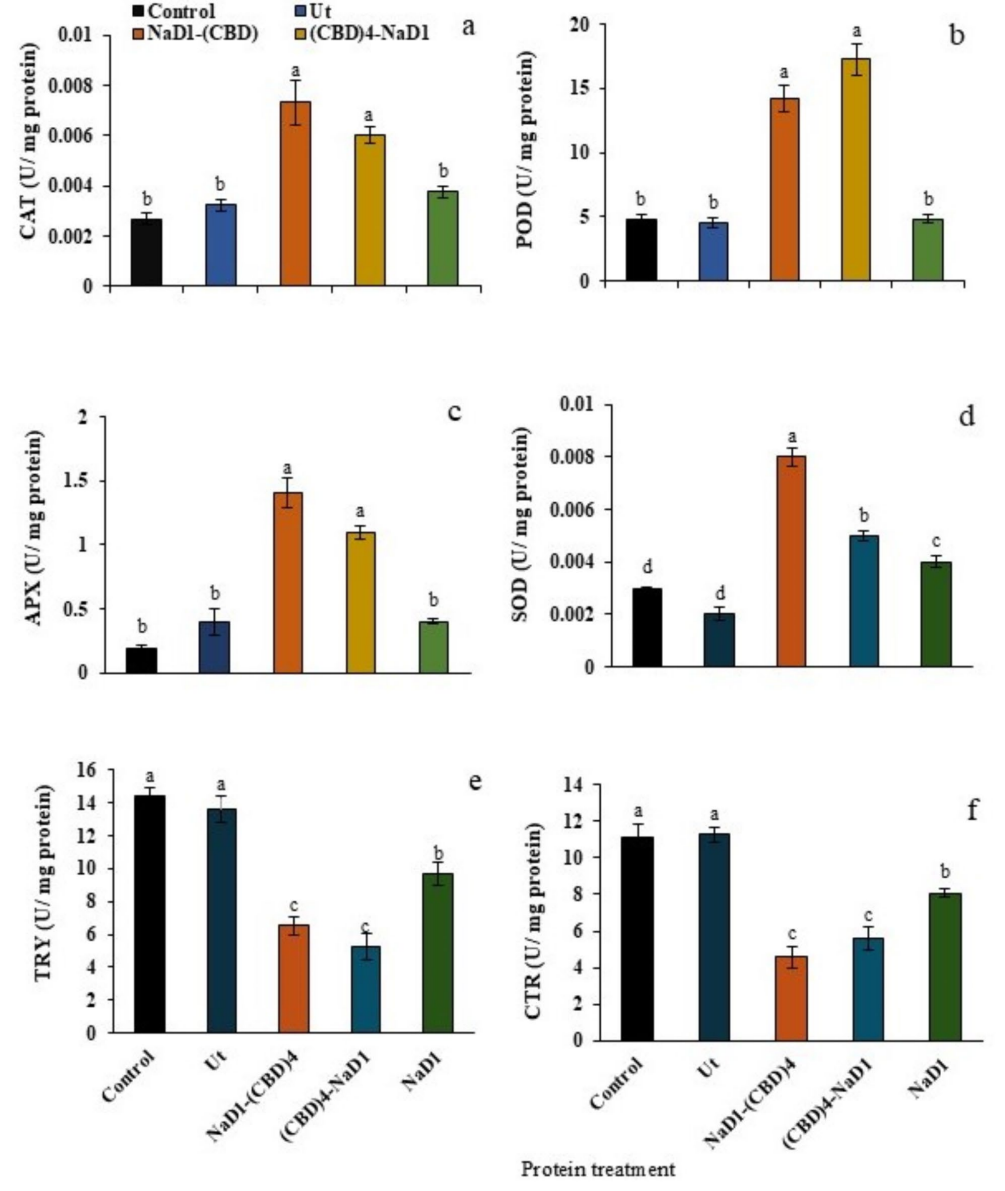
The activities of CAT, POD, APX, SOD, TRY and CTR enzymes in the third instar larvae of *C. suppressalis*. The feeding effects of the third instar larvae of *C. suppressalis* by 20 µg/ml five HR protein extracts including NaD1-(CBD)_4_, (CBD)_4_-NaD1, NaD1, non-transgenic (Ut) and the phosphate buffer as a negative control (Control) on CAT, POD, APX, SOD, TRY and CTR enzyme activities after 72 h.

After feeding of the third-instar larvae of *C. suppressalis* with 20 µg/ml HR protein extracts of NaD1-(CBD)_4_, (CBD)_4_-NaD1, NaD1, non-transgenic and phosphate buffer (control), the enzyme activity of CAT, POD, APX, SOD, TRY and CTR were measured after 72 h. Significant differences (*P* < 0.01) were observed among the five feeding treatments as shown in Supplementary Table S10. The comparison of enzyme activity among the six enzymes also revealed a significant difference (*P* < 0.01). While CAT activities in larvae fed by NaD1-(CBD)_4_ and (CBD)_4_-NaD1 were not significant, they differed significantly from NaD1, non-transgenic and phosphate buffer (*P* < 0.01) (Fig. 6a). POD activity in the larvae fed with NaD1-(CBD)_4_ and (CBD)_4_-NaD1 extracts was significantly elevated (*P* < 0.01) reaching the highest levels (three times more than NaD1) compared to the other extracts (Fig. 6b). In addition, APX activity in larvae fed with NaD1-(CBD)_4_ and (CBD)_4_-NaD1 increased significantly (56% and 44%) (*P* < 0.01) compared to others (Fig. 6c). SOD enzyme activity was highest in larvae fed by NaD1-(CBD)_4_, significantly differing from other protein treatments (*P* < 0.01). In contrast, SOD enzyme activity in larvae treated by NaD1, non-transgenic HR, and phosphate buffer was 1.6, 2, 4, and 2.6 times higher, respectively (Fig. 6d). The highest TRY and CTR activity was observed in the control group receiving phosphate buffer and non-transgenic HR extracts. TRY detected activity was lowest in the larvae fed by (CBD)_4_-NaD1, while CTR activity was found to be lowest in those fed by NaD1-(CBD)_4_ (Fig. 6e and f). The activity of these enzymes in NaD1 was higher than NaD1-(CBD)_4_ and (CBD)_4_-NaD1 (1.7 and 1.6 times, respectively). Remarkably, the activity of TRY and CTR in the larvae fed by phosphate buffer and non-transgenic HR extracts was almost twice compared to NaD1-(CBD)_4_ and (CBD)_4_-NaD1 (*P* < 0.01). Our findings suggest that larvae fed with recombinant proteins containing CBD exhibited higher antioxidant activity (CAT, POD, APX, and SOD) and lower TRY and CTR enzyme activity, compared to those fed with NaD1 lacking CBD. The increased antioxidant enzyme activity may indicate that larvae fed with CBD-containing recombinant protein extracts experienced higher stress conditions. Furthermore, the lower activity of TRY and CTR enzymes in these larvae may be attributed to the increased decomposition.

## Discussion

Various molecular biology techniques have been developed to boost plant resistance in response to diverse pathogens such as fungi and bacteria. These include production of proteins and metabolites involved in plant defense pathways, expressing pathogenesis-related proteins, and generating enzymes to break down fungal or bacterial cell walls^10^. AMPs as a part of the plant immune system are produced to defend plants against the harmful impact of microbial pathogens, particularly fungi^38^. Fungi severely threaten global food security, causing significant loss of agricultural products and destructive epidemics^39^. Defensins are the most important group of plant antifungal peptides^38^, and some of them have been extensively studied due to their potential ability in transgenic plants to protect against fungal diseases^40^. In this study, we enhanced the antifungal and insecticidal activities of the NaD1 peptide by fusing it with four chitin-binding domains (CBDs) from wheat germ agglutinin (WGA). Next constructs were expressed driven by 35 S promoter in tobacco HRs, leading to higher affinity towards chitin-containing fungal cell wall and midgut of pest insects.

The average inhibition rate of 50 µg ml^-1^ protein extracts on growth of *P. oryzae* mycelia on paper discs demonstrated that both NaD1-(CBD)_4_ and (CBD)_4_-NaD1 induced the highest inhibition rate with no significant difference (*P* < 0.01). Nevertheless, they displayed a significant difference (*P* < 0.01) in comparison to the protein extracts from NaD1 and non-transgenic HRs. Investigation of antifungal activity of the various concentrations of recombinant proteins on the initial fungal mycelial formation in the PDA medium during different time intervals resulted in similar responses to those of the disc diffusion method (Supplementary Table S7, Supplementary Table S8; Figs. 3 and 4). Therefore, NaD1-(CBD)_4_ and (CBD)_4_-NaD1 protein extracts are more effective and could considerably inhibit the growth of *P. oryzae*mycelium indicating that the presence of CBDs in both orientations of NaD1 can lead to equal antifungal activity. It can also be anticipated that NaD1 is effectively anchored to the fungal chitin cell wall through CBDs^14^. Chitin, a polymer made of N-acetylglucosamine, is a crucial component of the fungal cell wall and is present in all known fungi. This makes it a promising focus for the development of new therapeutic approaches^41^. The CBDs of WGA protein containing the amino acid sequences SXFGY or SXYGY (domains Hevein-like) can effectively inhibit the growth of chitin-containing fungi^42^. The fusion of CBDs to the NaD1 peptide increased the positive charge of recombinant proteins NaD1-(CBD)_4_ and (CBD)_4_-NaD1 from 12 to 25. The positive charge boosts NaD1 peptide interaction with negatively charged phospholipids in the cell membrane. AMPs’ positive charge is crucial for their interaction with negatively charged membrane phospholipids and can enhance their antimicrobial activity against pathogens^43,44^. Accordingly, the presence of CBD (with the protected sequence of hevein-like peptides) along with the NaD1 peptide could increase the stability of NaD1 peptide leading to the antifungal synergistic effect of recombinant proteins^10,11,19,20^. It has been also reported that classes of chitinase containing at least one CBD may lead to facilitating the catalytic activity of chitinases to digest bonds 1,4 -ß-linked N-acetyl-d-glucosamine in fungal pathogens^45^. The rate at which chitinase breaks down chitin and inhibits fungi increases with more contact between chitinase and its substrate on the fungal cell wall. Additionally, the level of exposure of chitin fiber bundles on the surface of the fungal cell wall is positively associated with the inhibitory activity of chitinase against fungi^19^. Therefore, it can be concluded that the recombinant proteins NaD1-(CBD)_4_ and (CBD)_4_-NaD1, with four CBDs (in this study) can bind to a larger surface area of the chitin structure of *P. oryzae*and deliver a stronger antifungal effect. Shams, et al^11^. fused the coding sequence of DrsB1 peptide to two repeats of CBD of *C. fulvum* Avr4 (CBD)2-DrsB1 resulting in enhancement of antifungal activity against chitin-producing *Alternaria alternata*and bacterial pathogens. They demonstrated that the enhancement antibacterial and antifungal potency of the (CBD)2-DrsB1 peptide in comparison to the DrsB1 likely results from a dual effect: an increase in the net positive charge, as well as an enhanced affinity to the fungal wall containing chitin following the addition of two copies of CBDs^11^. Also, Khademi et al., (2019) by fusing the coding sequence of DrsB1 with either the N-terminal or C-terminal of the CBD of the *Avr4* gene from *Cladosporium fulvum*demonstrated that CBD has a high affinity for cell wall components, which could enhance the accumulation of DrsB1 peptide on the surface of the fungal pathogen. This could result in a more efficient interaction between the positively charged recombinant peptides and the negatively charged membrane surface of the pathogens^10^. Badrhadad et al., (2018) fused the antibacterial peptide alfAFP to the CBD chitinase-encoding gene of rice, and then introduced to *N. tabacum* resulting in enhancement of resistance against *Fusarium solani*^17^. This resistance is attributed to the binding of CBD to cell wall chitin, chitin oligomers, and N-acetylglucosamine, which allows the alfAFP peptide to access the fungal cell wall and delay the onset of the disease in transgenic lines^17^.

In this study, drawing on the achievements of previous researchers, we employed and synthesized the NaD1 peptide both independently and in conjunction with the CBDs (functional domains) of the WGA protein in tobacco HRs, which has not previously been leveraged in the development of transgenic plants. The recombinant proteins containing CBD demonstrated superior antifungal properties and enhanced resistance to the *P. oryzae* fungal pathogen compared to those lacking CBD. Accordingly, binding CBD to NaD1 probably leads to (1) increased half-life of the active NaD1 peptide, (2) enhanced chitin affinity, breaking down fungal wall structure and increasing susceptibility to antifungal properties, (3) heightened cationic charge of the NaD1 peptide, enhancing interaction with negative phospholipids like PIP_2_or PA in the membrane, and (4) increased antifungal activity due to the presence of protective domains Hevein-like (SXFGY or SXYGY) in CBD-A and CBD-B. Moreover, the accumulation of NaD1 peptide on fungal pathogens using the Carpet-like method disrupts fungal cells and causes cell death^46^. Consequently, fusion of four CBDs of WGA protein to NaD1 peptide likely increases the affinity of the recombinant protein for the fungal cell wall chitin and increases its ability to inhibit the growth of *P. oryzae* fungal mycelia. This innovative approach holds great promise for the development of transgenic crops with improved disease resistance, ultimately leading to higher crop yields and reduced reliance on chemical pesticides. Our findings contribute to the ongoing efforts to enhance food security and sustainability in agriculture.

In addition to antifungal activity, NaD1 displayed insecticidal activity. The analysis of variance and mean comparisons on the survival percentage of third-instar larvae of *C. suppressalis* feeding by protein extracts NaD1-(CBD)_4_ and (CBD)_4_-NaD1 (20 µg/ml) displayed the highest mortality rate after 48 and 72 h .Therefore, the insecticidal effect of protein extracts becomes visible after 48 h (Fig. 5). NaD1 reduced the growth of Lepidopteran pests such as *Helicoverpa armigera* and *Helicoverpa punctigera*when expressed in an artificial diet or transgenic plants^29^. NaD1 suppresses the activity of key digestive enzymes TRY and CTR, which are vital for the *Helicoverpa*pest^29^. We also found a clear reduction in TRY and CTR activity in the third- instar larvae of *C. suppressalis* after feeding by protein extracts NaD1-(CBD)_4_, (CBD)_4_-NaD1 and NaD1 (Fig. 6). When *NaD1* (by inhibiting serine proteases such as TRY and CTR) was introduced into Royal Gala apples via *Agrobacterium*-mediated transformation, Apple moth larvae consuming NaD1-containing leaves lost their weight and pupae size compared to the control group as well as displayed morphological changes in adults^30^. Among carbohydrate-binding modules, CBM18, which has been found in several plants and fungi, binds to chitin N-acetyl-D-glucosamine monomers^47^. Plant lectins, especially chitin-binding lectins, belong to the CBM18 group and are composed of cysteine-rich amino acid sequences called CBD such as WGA wheat and include four types of CBD^31^. These CBDs can interact with the chitin network of the peritrophic wall of an insect’s digestive system. The peritrophic membrane consists of a chitin fiber network and acts as the lining in an insect’s midgut, physically protecting the midgut cells. The change in the morphology of the European corn borer peritrophic wall was found after feeding on a diet containing WGA from CBM18 group^48,49^. In this research, by adding four CBDs of WGA to NaD1 peptide, we observed more insecticidal activity (~ 30%) compared to the peptide without CBD. Moreover, a clear increase in *C. suppressalis* larvae’s death rate could probably be due to the proper binding and deploying of NaD1-(CBD)_4_ and (CBD)_4_-NaD1 proteins on tissues containing chitin. Exploring the molecular mechanism of the interaction of the NaD1 peptide and CBDs of the WGA protein could offer valuable insights for creating new insecticidal agents and their potential use in pest control. Understanding these interactions at a molecular level could pave the way for the development of novel insecticidal agents with enhanced efficacy and specificity.

The antioxidant defense is the most crucial and efficient internal defense system against stressful factors^50^. Antioxidant enzymes demonstrate adverse environmental effects^51^and shield organisms against oxidative stress by scavenging reactive oxygen species (ROS)^52,53^. Insecticides induce oxidative stress, which can be assessed by analyzing antioxidant enzyme activities^54^. Antioxidant enzymes such as SOD, APX, CAT, and POD protect organisms from oxidative stress by scavenging ROS. SOD is the main defense enzyme against superoxide radicals, catalyzing their conversion into H_2_O_2_ and O_2_^52^, while CAT, APX, and POD eliminate H_2_O_2_^53^. Hereby, we measured the activity of these four enzymes in the third instar larvae of *C. suppressalis* after feeding them by various HR protein extracts and compared them with the control once. The activity of antioxidant enzymes CAT, POD, APX, and SOD in the larvae fed by NaD1-(CBD)_4_ and (CBD)_4_-NaD1 transgenic extracts remained much higher in comparison with the others. While, CAT, APX and POD activity was not significantly different amongst NaD1, non-transgenic HR extract, and phosphate buffer (control). Moreover, the activity of SOD in the larvae fed by NaD1 transgenic extracts stayed higher than in the larvae fed with the non-transgenic HR extract, and phosphate buffer (Fig. 6). Insects utilize three main enzymes, SOD, CAT, and POX to combat oxidative stresses and uphold their antioxidant defenses^55^. Feeding Galleria mellonella larvae with Azadirachtin extract, a natural insecticidal compound from neem tree seeds, significantly boosts the activity of antioxidant enzymes after 72 h^56^. The potential impact of three components - α-pinene, trans-anethole, and thymol (botanical insecticides) on the antioxidant system of *Ephestia kuehniella*Zeller was examined^52^. The treated larvae with an artificial diet containing each of these compounds showed a significant increase in the activities of SOD, POX, and CAT^55^. Our study demonstrated that adding CBDs to NaD1 accompanied by enhanced activity of antioxidant enzymes likely induce a stress condition, in association with the enhanced level of ROS in the larvae. Therefore, CBD, in addition to providing a better connection for recombinant proteins on chitinous tissue, its insecticidal activity probably leads to an enhanced level of ROS in the larvae^20,57^.

The increase in antioxidant enzyme activity of SOD, APX, CAT, and POD, as a result of the presence of CBDs, might be associated with the enhancement in ROS biosynthesis resulting in the induction of a stressful condition in the larvae. When excessive levels of reactive oxygen species (ROS) accumulate in plant cells, they are harmful to the structure of proteins, nucleic acids, lipids, membranes, and organelles. This, in turn, triggers the activation of cell death processes such as apoptosis^58^. Consequently, apoptosis is probably triggered in the insects fed by two NaD1-(CBD)_4_ and (CBD)_4_-NaD1 recombinant proteins in response to the stressful conditions. *Lacanobia oleracea* larvae, when nourished with an artificial diet incorporating 2% *Galanthus nivalis* (Lectin), exhibited a diminished survival rate in contrast to the control group. This outcome was attributed to the induction of apoptosis by *Galanthus nivalis*lectin, as reported by Rahimi, et al^59^.. Therefore, the inclusion of CBD of WGA lectin in NaD1-(CBD)_4_ and (CBD)_4_-NaD1 recombinant proteins may result in elevated ROS production, leading to cell death, ultimately increasing the mortality rate of *C. suppressalis* larvae.

The larvae fed by the protein extract (CBD)_4_-NaD1 and NaD1-(CBD)_4_ in turn displayed the lowest level of TRY and CTR enzyme activity. Parallel to the obtained results, the activity of TRY and CTR enzymes in larvae fed by rice stalks dipped in phosphate buffer (control) and non-transgenic HR extract also displayed the highest TRY and CTR activity (Fig. 6e and f). Therefore, presence of NaD1 and CBDs in protein extracts of NaD1-(CBD)_4_ and (CBD)_4_-NaD1 could probably induce an intense stressful condition in the larvae in association with more ROS biosynthesis when compared with the control once confirming the increase of anti-insect or insecticidal properties of these two recombinant proteins. It has already been found that NaD1 peptide inhibits the activity of essential enzymes, including TRY and CTR in *Helicoverpa*leading to larvae mortality^29^. Similarly, the light-brown apple moth fed on the apple leaves expressing NaD1 showed an apparent damage and mortality^30^. It was discovered that *Helicoverpa armigera*larvae feeding on transgenic tobacco leaves expressing NaD1 (NA-PI) inhibiting TRY and CTR showed increased mortality and slower larval development compared to larvae fed non-transgenic tobacco^60^. When proteinase inhibitors from *N. alata*were expressed in tobacco, they enhanced plant resistance to Lepidoptera, Coleoptera, Orthoptera, and Diptera pests by inhibiting TRY and CTR. The NaD1 peptide exhibited 37–79% inhibition on TRY and CTR enzyme activity^61^. However, in the current study, the activity of TRY and CTR enzymes was decreased in the larvae fed on the extracts containing NaD1-(CBD)_4_ and (CBD)_4_-NaD1 than NaD1 proteins, suggesting CBD may play a role in increasing the activity of recombinant proteins and consequently a higher rate of larvae mortality. Hence, due to their better interaction with chitinous midgut tissues of *C. suppressalis* larvae, they could highly inhibit TRY activity and CTR. The decrease in the activity of TRY and CTR in the larvae fed with NaD1-(CBD)_4_ and (CBD)_4_-NaD1 can be attributed to the increase in their decomposition. On the other hand, Lectin (such as WGA, the source of CBDs in this study) treatment leads to cytotoxicity in the epithelial cells of *H. armigera*midgut^59^. Lectins also decrease the activity of digestive enzymes such as TRY and CTR by binding to glycan receptors at gut epithelial cells, causing cytotoxicity and fewer active cells to secrete enzymes^59^. Hence, the fusion of CBDs and NaD1 could probably alter TRY and CTR activity by better placement of NaD1 (in NaD1-(CBD)_4_ and (CBD)_4_-NaD1) on chitinous tissue. Accordingly, biosynthesis of two recombinant proteins NaD1-(CBD)_4_ and (CBD)_4_-NaD1 in crop plants and subsequently generation of resistant lines against some destructive fungal pathogens and pest larvae could be very fruitful. These findings could have significant implications for the agricultural industry, as the ability to create resistant crops could greatly reduce the need for harmful pesticides and other chemicals. Additionally, the use of recombinant proteins offers a promising avenue for developing more sustainable and environmentally friendly agricultural practices. Further research in this area could lead to development of a wide range of new crop varieties with enhanced resistance to a variety of pests and pathogens, ultimately improving food security.

## Conclusions

This study discusses the potential of new recombinant antimicrobial peptides, specifically NaD1 peptide fused to and chitin-binding domains (CBDs) of WGA lectins, in controlling fungi and insect pests in agriculture. Compared to chemical pesticides, AMPs offer a safer and more sustainable solution due to their lower side effects. Our study confirmed the effectiveness of overexpressing NaD1 fused with four CBDs of WGA protein in tobacco plants against *P. oryzae* fungi and *C. suppressalis* larvae. Results of this study suggest that the fusion protein enhances antifungal and insecticidal activity, possibly by aiding peptide penetration into cell membranes. Utilizing these natural compounds in transgenic plants could improve crop yields and overall plant health while reducing reliance on chemical pesticides. Further research will explore the mechanisms behind their efficacy, aiming to develop more targeted and environmentally friendly pest control methods.

### Methods

#### Designing and constructing of the recombinant cassettes

The coding sequence of NaD1 antimicrobial peptide from *N. alata* (GenBank accession number: AF509566.1) including a 141 nucleotide-long DNA was fused either to the C- or N-terminus of a 513 nucleotide‐long sequence encoding the four CBDs of the WGA protein from *Triticum aestivum* (GenBank accession number: M25536.1), by a linker sequence. After determination of the linker length by *Modeller*_10.2_ software, the linker (EAAAK)_2_sequence^62^ was fused to *NaD1* and *CBD* (see Determination of the linker length Section). Three recombinant constructs including *NaD1-(CBD)*_*4*_, *(CBD)*_*4*_*-NaD1*, and *NaD1* were designed to be synthesized (Fig. 1). The coding sequence of the signal peptide (SP) of the *vicilin* gene (GenBank accession number: Q702P1) consisting of 15 amino acids from *Pisum sativum*was also fused to the 5′ terminal of the three constructs to ensure secretion of recombinant proteins into the apoplastic (intercellular) space^2^. The Polyhistidine Tag (His)_6_ sequence (HT) was engineered to the N-terminal after the signal peptide for recogntion of the recombinant proteins (Fig. 1). Furthermore, the cleavage sites of *Nco*I and *Bam*HI restriction enzymes were designed at both ends of 5′ and 3′ of the recombinant genes for cloning objectives (Fig. 1). Finally, three recombinant constructs including pUC57: *NaD1- (CBD)*_*4*_, pUC57: *(CBD)*_*4*_*- NaD1*, and pUC57: *NaD1* were chemically synthesized and cloned into the pUC 57 cloning vectors by ShineGene company, China. Then, the recombinant plasmids were transferred to the *E. coli* strain *DH5α*using the heat shock method^63^.

### Determination of the linker length and assessment of the antimicrobial sequence

The possible connection of WGA protein (containing CBD) (PDB code: 4AML, 171 amino acids) to the NaD1 peptide (PDB code: 1MR4, 47 amino acids) was evaluated by two modes: (1) The direct connection and (2) The connection via a linker. For the second mode, three types of linkers, including 2, 4, or 6 repetitions of EAAAK, were considered. Also, for binding, two possible directions including (CBD)_4_-NaD1, and NaD1-(CBD)_4_ were considered, and the resulting proteins were modeled by Modeler _10.2_ software (Supplementary Fig. S1).

The designed cassettes were evaluated for their antimicrobial properties using different algorithms provided by the Collection of Antimicrobial Peptides Database (CAMP), such as the Support Vector Machine (SVM) classifier, Random Forest Classifier, and Artificial Neural Network (ANN) classifier. The designed sequences were compared with other antimicrobial sequences to predict the probability of their antimicrobial activity. Next, the functional domains were checked using the Pfam database, and the physical and chemical properties of the sequences were evaluated by ProtParam database algorithms (https://www.expasy.org/resources/protparam*)* before chemical synthesis. After codon optimization of cassettes based on codon usage bias of *N. tabacum* by the Genscript database, *Nco*I and *Bam*HI cleavage sites were inserted at the 5’ and 3’ ends of the sequences. Then, based on the Novoprolabs site, the amount of Codon Adaptation Index (CAI) before and after optimization (more than 0.8), and the amount of GC (30–70%) were checked (https://www.novoprolabs.com/tools/codon-optimization).

### ***E. coli ***recombinant plasmid extraction

The plasmids of pUC57: *NaD1- (CBD)4*, pUC57: *(CBD)4- NaD1*, and pUC57: *NaD1* were extracted from *E.coli DH5α* following the instructions of Roch High Pure Plasmid Isolation Kit. Then, the recombinant vectors and expression plasmid pGSA1285 were digested by *Bam*HI and *Nco*I following the instructions provided by Thermo Fisher Scientific at 37 °C, and finally, the reaction was deactivated at 80 °C. Next, the *NaD1- (CBD)4*, *(CBD)4- NaD1*, and *NaD1* fragments and digested expression plasmid pGSA1285 were purified using the DNA extraction kit from GeneJET Gel Extraction Kit - Thermo Fisher Scientific. A ligation reaction between each one of *NaD1- (CBD)4*, *(CBD)4- NaD1* (Both 782 bp) and *NaD1* (239 bp) with the digested vector pGSA1285 (9553 bp) was performed using the Sinaclon T4 DNA Ligase enzyme. The ligation reaction was incubated at 22 °C for 2 h, and at the end, the enzyme was deactivated at 64 °C for 15 min. Then, the recombinant expression plasmids pGSA1285: *NaD1- (CBD)4*, pGSA1285: (*CBD)4- NaD1*, and pGSA1285: *NaD1* were transferred to *E. coli DH5α*competent cells, using the heat shock method^63^. The clones were grown at 37 °C on a solid LB (Luria broth) culture medium containing 34 µg/ml of chloramphenicol antibiotic for 24 h, followed by their evaluation for the presence of the fragments.

### Verification of gene cloning in pGSA1285 and transformation of ***Agrobacterium rhizogene***s

To confirm the presence of desired gene fragments in pGSA1285, a colony PCR approach was employed, using the specific primers for the *NaD1- (CBD)4*, *(CBD)4- NaD1*, and *NaD1* (Table 1 and Supplementary Table S6). The PCR products were visualized on a 1.5% agarose gel. Restriction analysis was performed using *Bam*HI and *Nco*I cleavage sites, present on both sides of the recombinant fragments, at 37 °C following Thermo Fisher Scientific instructions (Fig. 1e). To verify the accuracy of the sequences, lack of nucleotide mutations and correct fusion of gene fragments in pGSA1285, Sanger sequencing was carried out (Pishgam and Applied Biosystems, Iran). Subsequently, correct sequences of the recombinant plasmids were confirmed using SnapGene software and the Clustal Omega program available on the EMBL (https://www.ebi.ac.uk/Tools/msa/clustalo/). Then, the recombinant plasmids were transferred to *Agrobacterium rhizogenes* strain *ATCC 15,834*by the thawing and freezing method^63^. A colony PCR reaction was conducted on the recombinant Agrobacterium colonies grown on the selective culture medium containing chloramphenicol (50 g/ml), and rifampicin (50 g/ml) using *NaD1- (CBD)4*, *(CBD)4- NaD1*, and *NaD1* genes-specific primers. The recombinant genes in HRs were expressed under the control of the (3X) 35 S cauliflower mosaic virus promoter (Fig. 1a).

### Plant materials

The seeds of *N. tabacum* L. var Xanthi were provided by Tirtash Educational and Research Center, Behshahr, Mazandaran, Iran. The seeds were surface sterilized in 70% ethanol (v/v) for 60 S and then soaked in 5% sodium hypochlorite for 10 min. The seeds were rinsed three times with sterile distilled water. The sterile seeds were grown on the MS solid medium (30 g l^−1^ sucrose, 6.5 g l^−1^agar) for one month at 25 ± 1 °C with a 16/8 h light/dark photoperiod^10,11,14^.

### ***Agrobacterium rhizogenes***-mediated Transformation and HR development

The *N. tabacum*L. cv. Xanthi leaf discs (1 cm^2^) were pre-cultured on MS medium overnight, then they were separately co-cultured with the *A. rhizogenes* (ATCC15834) suspension culture containing the pGSA1285: *NaD1- (CBD)*_*4*_, pGSA1285: *(CBD)*_*4*_*- NaD1* or pGSA1285: *NaD1* expression vectors for 5–7 min gently shaking. The leaf discs were air dried and transferred to an MS solid medium containing 50 g l^−1^ sucrose at a pH of 5.7 ± 1 at 24 ± 1 °C in darkness for 48 h. Subsequently, the leaf discs were transferred to a new MS medium, containing 500 mg l^−1^cefotaxime and 50 mg l^−1^ kanamycin and maintained at 24 ± 1 °C under a 16/8 h light/dark photoperiod for 8–10 days. Next, the freshly grown HRs were transferred to the new MS medium containing 400 mg l^−1^cefotaxime and 75 mg l^−1^ kanamycin, for 10–12 days. Then the HRs were transferred to the fresh liquid MS medium containing 400 mg l^−1^ cefotaxime and 100 mg l^−1^ kanamycin at 24 ± 1 °C in the dark, and HRs were sub-cultured every 2 weeks. non-transgenic HRs were produced by transforming leaf discs with *A. rhizogenes*^64^.

### Screening the transgenic hrs and gene expression analysis

Genomic DNA was extracted from the putative transgenic *NaD1-(CBD)*_*4*_, *(CBD)*_*4*_*-NaD1*, *NaD1*HRs lines, and the non-transgenic HR, using the CTAB method^65^. Using *VirG*-specific primers and *rolC* specific primers, the presence of the T-DNA and absence of *A. rhizogenes* in HRs were confirmed (Table 1 and Supplementary Table S6). Finally, to screen the transgenic HR clones, the *NaD1-(CBD)*_*4*_, *(CBD)*_*4*_*-NaD1*, and *NaD1* specific primers were used (Table 1). The PCR reaction temperature schedule is shown in Supplementary Table S6. The amplified fragments of the PCR reactions are presented in Fig. 1.

For gene expression analysis of *NaD1-(CBD)*_*4*_, *(CBD)*_*4*_*-NaD1*, and *NaD1*, RNA was isolated from the transgenic and non-transgenic HRs using the DENAzist RNA isolation kit. Agarose gel electrophoresis (1%) and Thermo ScientificTM nanodrop were applied to determine the quality and quantity of extracted RNA. After treatment of total RNA with DNase I, the cDNA synthesis was performed using the CinaClon First Strand cDNA Synthesis Kit (CinaClon, Iran). In order to verify the correctness of cDNA synthesis and evaluation of the expression of *NaD1-(CBD)*_*4*_, *(CBD)*_*4*_*-NaD1*, and *NaD1* genes in HRs and non-transgenic HRs, a semi-quantitative RT-PCR reaction was conducted, using the housekeeping control gene *elf1*α primers (177 bp) (Fig. 1c), and *NaD1-(CBD)*_*4*_, *(CBD)*_*4*_*-NaD1*, and *NaD1* specific primers (Table 1 and Supplementary Table S6), followed by visualizing and photographing the reaction products by electrophoresis on a 1.5% agarose gel (Fig. 1d).

### ELISA and western blot analysis of the recombinant proteins

The transgenic and non-transgenic HRs tissues were gently homogenized using liquid nitrogen in 1:1 (w/v) cold buffer (phosphate buffer 50 mM, pH: 7, containing 1 mM phenylmethylsulfonyl fluoride (PMSF) protease inhibitor (Sigma-Aldrich)^10,11^. Then vortexed for 5 min; samples were centrifuged at 13,000 rpm for 30 min at 4 °C, the upper phase was separated and filtered by a 0.45-micron filter, and the protein concentration was evaluated by the Bradford method^66^.

Then, sodium dodecyl sulfate-polyacrylamide gel electrophoresis or 12% SDS-PAGE (12% separating gel and 5% Stacking gel) was prepared by Mini-PROTEAN Tetra Vertical Electrophoresis Cell (Bio-Rad) to check the quality and presence of protein bands containing NaD1-(CBD)_4_, (CBD)_4_-NaD1 (~ 27 kDa), and NaD1 (~ 9 kDa) peptides. Coomassie Brilliant Blue (R-250) was used for staining^67^.

Production of recombinant proteins (His)6-NaD1-(CBD)_4_, (His)6-(CBD)_4_-NaD1, and (His)6-NaD1 in the total protein extracts of HRs was checked by ELISA method. Proteins of transgenic and non-transgenic HRs were extracted according to the instructions of the His-Tag Protein ELISA kit, catalog number AKR-130 of Cell Biolabs, Inc., and were read at a wavelength of 450 nm by the microplate reader of Awareness Technologies Inc. Stat Fax 4200.

Moreover, to detect the presence of NaD1-(CBD)_4_, (CBD)_4_-NaD1, and NaD1, the protein extracts (10 µg each) were loaded and separated on the SDS-PAGE gel, transferred to a polyvinylidene fluoride (PVDF) membrane (Roche, UK) followed by a blocking procedure with 3% BSA in PBS phosphate-buffered saline for 1 h. The membranes were incubated at 4 °C with 1:200 dilution of the mouse anti-His (sc-8036) antibody (Santa Cruz Biotech) overnight. The secondary m-IgG1 BP-HRP Bundle (sc-531885) antibody (Santa Cruz Biotech) was used to detect the antibody-antigen complex on the membranes. The blots were developed with a chemiluminescence detection system (Pierce ECL, Thermo Fisher Scientific). For that, HyBlot CL (Denville Scientific, Metuchen, NJ) and Amersham Biosciences Hyperfilm were utilized to detect multiple proteins, the membranes were then stripped and reprobed. Immunoblots were quantified by the imageJ 1.62 software.

### Antifungal activity assay

#### Investigation of the antifungal effect of recombinant proteins on the initial fungal mycelial formation

The total proteins extracted from transgenic HRs were prepared at final concentrations of 20, 50, and 100 µg ml-1. Subsequently, these proteins were incorporated into sterile PDA (Ibresco) medium at 55 °C. Once the PDA medium with the recombinant proteins had solidified and cooled to 4 °C, 10 mm diameter plugs, which were taken from a 4-day-old *P. oryzae* fungal culture, provided by Dr. Sedigheh Mousanejad, Department of Plant Protection, The University of Guilan were inoculated onto the center of petri dishes, containing PDA medium and the recombinant proteins. Each experiment was carried out in triplicate.

The petri dishes were incubated at 25 ± 1 °C in darkness. The growth of the fungus was monitored from the 2nd to the 10th day post-inoculation. To evaluate the inhibition of mycelial growth, the diameter of the fungal growth circle surrounding the central disk was measured in millimeters in two directions and compared to the control. The percentage of mycelium growth inhibition was calculated using the following equation:

$${\text{PI}}(\% ) = ({\text{DC}} - {\text{DT}})/{\text{DC}}*100$$

Where PI demonstrates the percentage of inhibition of mycelium growth, DC shows the radial growth zone diameter in control, and DT displays the diameter of the radial growth zone in the treatment^10,11,68^.

#### Investigation of the antifungal effect of recombinant proteins by the hyphal extension-inhibition assay

Hyphal extension-inhibition assay was applied to determine the antifungal activity of recombinant proteins under sterile conditions^69^. Fungal mycelia from actively growing *P. oryzae* plates were positioned at the center of petri dishes containing PDA medium (Ibresco) for cultivation. Following an incubation period at 25 ± 1 °C for 48–72 h, sterile filter paper discs were positioned in front of the advancing fungal mycelium. Subsequently, 20 µl of the extracted proteins (concentration: 50 µg ml^−1^) were applied to each disc. The plates were then further incubated at 25 ± 1 °C for 72 h, with the inhibition of mycelial growth being monitored and recorded every 24 h. The mycelium growth area on each disc was quantified using imageJ software at 24, 48, and 72 h post-disk placement, with each disc having a defined surface area of 28.27 mm^2,69^.

#### Insecticidal activity assay

Egg clusters of *C. suppressalis*, obtained from the fields of the Iranian Rice Research Institute in Amol, Iran, which had not been exposed to any insecticides, were collected. Upon arrival at the laboratory, parasitized larvae of *C. suppressalis*were isolated and reared in sterile culture tubes under control conditions of 28 ± 1 °C temperature, 70–80% relative humidity, and a 16/8 h light/dark photoperiod. These larvae upon hatching, were fed on rice stems of Hashemi cultivar within the laboratory until they reached the the third instar. Subsequently, the larvae were provided with fresh stems of Hashemi rice variety in cleaned rearing containers daily for continued growth and development^70^.

The resistance of *C. suppressalis* populations to NaD1-(CBD)_4_, (CBD)_4_-NaD1, and NaD1 recombinant proteins was assessed using the seedling dip method as outlined by^71^. Rice stems from the susceptible Hashemi cultivar, grown without exposure to insecticides, were trimmed to a length of 6 cm at the rice tillering stage. Total protein extracts from HR lines containing the recombinant proteins NaD1-(CBD)_4_, (CBD)_4_-NaD1, and NaD1 were prepared at a final concentration of 20 µl for bioassay and determination of insecticidal properties.

The rice stems were immersed in each extract for a duration of 2 h and then air-dried until no residual moisture remained on the surface. A petri dish with a diameter of 9 cm, containing four moist filter papers, was prepared to accommodate seven stem sections. Subsequently, five third-instar larvae were introduced to each petri dish for the bioassay and evaluation of insecticidal effects^70^.

Each treatment group was replicated three times, with negative control stems treated with a phosphate buffer. Assessments of mortality were conducted at 24, 48 and 72 h post-treatment. Larvae were classified as deceased, if they displayed an inability to move normally when gently stimulated with a soft brush^70^.

#### Enzymatic assays

For enzymatic assays, larvae exposed to total recombinant protein extracts were harvested after 72 h. The collected samples underwent homogenization using the Qiagen TissueLyser LT Bead Mill in 300 µl of Tris buffer (pH: 7) for 30 s, followed by centrifugation at 8000 rpm and 4 °C for 20 min. Supernatants were collected as the crude enzyme source. Enzyme activities were assessed using optical spectrometry with a Biochrom, Libra S22 spectrophotometer and a microplate reader (Awareness model Technology Inc.). The DNAbiotech kit was utilized to quantify the total protein concentration according to the Bradford method^66^.

Catalase activity (CAT, EC. 1.11.1.6) was evaluated by measuring the decrease in light absorption at 240 nm, using a spectrophotometer. Changes in absorbance over time within a minute were recorded to determine enzyme activity^72^.

Spectrophotometric absorbance readings at 430 nm were taken every 30 s over 2 min to quantify peroxidase enzyme activity (POX, EC. 1. 11. 1.7) based on the method of Addy and Goodman^73^.

Ascorbate peroxidase activity (APX, EC 1. 11. 1. 11) was determined by measuring the extent of of ascorbate oxidation at 290 nm over 5 min, using a spectrophotometer^74^.

Superoxide dismutase activity (SOD, EC 1.15.1.1) was assessed by measuring optical absorbance at 560 nm^75^.

Trypsin enzyme activity (TRY, EC 3.4.21.4) was determined using a 1 mM concentration of BApNA (N Benzoyl-DL-arginine-p-nitroanilide) as a specific substrate at 405 nm^76^.

Chymotrypcin enzyme activity (CTR, EC 3.4.21.1) was measured at 405 nm using a 1 mM concentration of SAAPPpNA (N-succinyl-alanine-alanine-proline-phenylalanine-p-nitroanilide) as a specific substrate^76^.

### Statistical analysis

To analyze the data variance, a one-way ANOVA (Analysis of variance) was executed using SAS 9.1 software, with a significance level set at 0.01. Post-hoc analysis of mean comparisons was carried out using the Least Significant Difference (LSD) test. The experimental design followed a completely randomized design with each treatment group replicated three times. For visualization of the experimental results, graphs were generated, using Excel 2016.

## Electronic supplementary material

Below is the link to the electronic supplementary material.

Supplementary Material 1

## Data Availability

All data generated or analysed during this study are included in this published article (and its Supplementary Information file).
