# Supplementary material for: Expression of a novel NaD1 recombinant antimicrobial peptide enhances antifungal and insecticidal activities
Source: Sci Rep. 2024 Oct 5;14:23235. doi: 10.1038/s41598-024-73710-3 (PMC11455875; doi:10.1038/s41598-024-73710-3)
Supplement: Supplementary file 1 — Supplementary Material 1 [file 41598_2024_73710_MOESM1_ESM.pdf]

Journal: Scientific reports

Supplementary information

Expression of a novel NaD1 recombinant antimicrobial peptide enhances antifungal and insecticidal activities

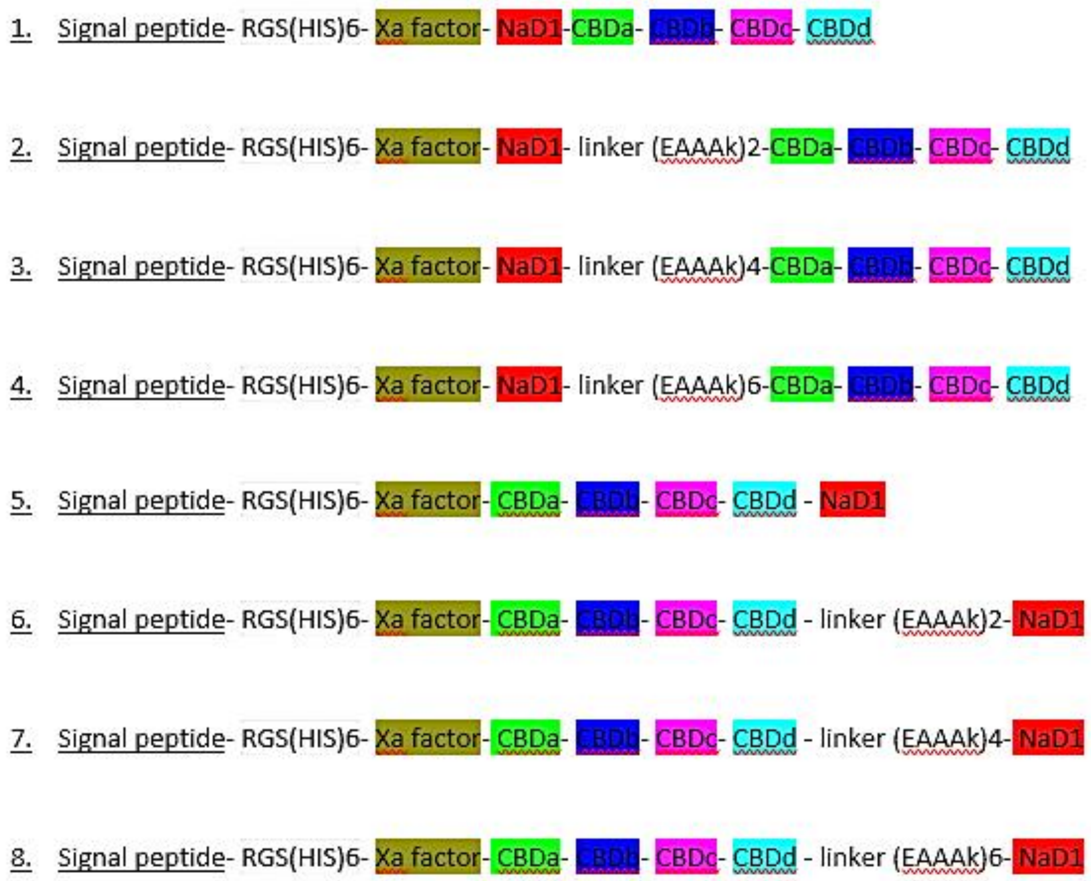

Figure S1. Different modes of recombinant protein to check the appropriate length of the linker for the recombinant protein

Modes 1-4 are structures with different lengths of the EAAAK linker sequence in the mode where chitin-binding domains are in the C-terminal of the NaD1 peptide, Mode 5–8 structures with different lengths of the EAAAK linker sequence in the mode where chitin-binding domains are at the N-terminal of the NaD1 peptide.

Proteins	Without Linker		Linker					
			2 Repeat		4 Repeat		6 Repeat	
	CBD-NaD1	NaD1-CBD	CBD-NaD1	NaD1-CBD	CBD-NaD1	NaD1-CBD	CBD-NaD1	NaD1-CBD
**4AML**	0.95	0.92	0.92	0.99	0.98	0.95	0.99	0.96
**1MR4**	0.98	0.99	0.99	0.98	0.98	0.99	0.99	0.98

Table S1. Determining the length of EAAAK linker based on *Modeller* software

Seq. ID.	Class	AMP Probability
NaD1-(CBD)4	AMP	1.000
$(CBD)_4$- NaD1	AMP	1.000
NaD1	AMP	0.978

Table S2. The result of investigating the antimicrobial properties of the NaD1-(CBD)4, NaD1 -(CBD)4 and NaD1 cassettes by the Support Vector Machine (SVM) classifier algorithm

Seq. ID.	Class	AMP Probability
NaD1-(CBD)4	AMP	0.7875
$(CBD)_4$- NaD1	AMP	0.799
NaD1	AMP	0.8935

Table S3. The result of investigating the antimicrobial property of NaD1-(CBD)4, NaD1 -(CBD)4 and NaD1 cassettes by Random Forest Classifier algorithm

Seq. ID.	Class
NaD1-(CBD)4	AMP
$(CBD)_4$- NaD1	AMP
NaD1	AMP

Table S4. the result of investigating the antimicrobial property of NaD1-(CBD)4, NaD1 -(CBD)4 and NaD1 cassettes by the Artificial Neural Network (ANN) classifier algorithm

Seq. ID.	Class	AMP Probability
NaD1-(CBD)4	AMP	1.000
$(CBD)_4$- NaD1	AMP	1.000
NaD1	AMP	0.974

Table S5. The result of investigating the antimicrobial property of NaD1-(CBD)4, NaD1 -(CBD)4 and NaD1 cassettes by the Discriminant Analysis classifier algorithm

Step	Temperature (C°)	Time	Cycles
Initial denaturation	95	5 min	
Denaturation	95	45 Sec	35
Annealing	Table 1	30 Sec	
Extension	72	30 Sec	

Final extension	72	7 min

Table S6. PCR, colony PCR and RT-PCR reaction program for all genes

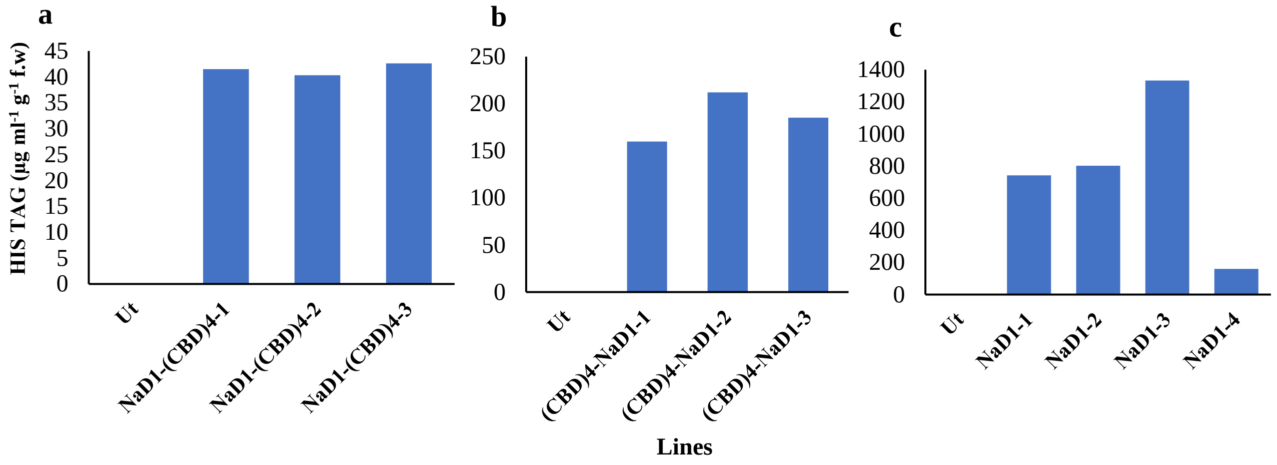

Figure S2. Production of the recombinant protein containing Histidine tag sequence, $(His)_6$ by ELISA test.

The recombinant peptides were identified using a His-Tag Protein ELISA kit (AKR-130 of Cell Biolabs, Inc.). Transgenic HRs (**a**) expressed $(His)_6$-NaD1-$(CBD)_4$; (**b**) expressed $(His)_6$- $(CBD)_4$- NaD1,(**c**) Transgenic HRs expressed $(His)_6$-NaD1 peptide. Ut: represents non-transgenic HRs.

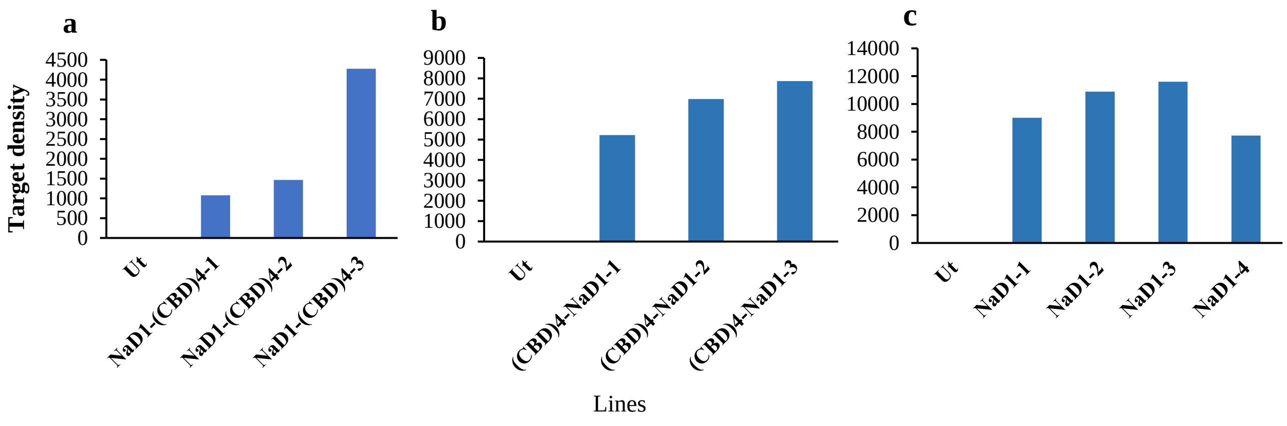

Figure S3. Quantification of immunoblots using the image j 1.62 software.

The recombinant peptides were identified by utilizing the Anti-His monoclonal antibody sc-8036 (Santa Cruz Biotech). Transgenic HRs (**a**) expressed $(His)_6$-NaD1-$(CBD)_4$; (**b**) expressed $(His)_6$- $(CBD)_4$- NaD1,(**c**) Transgenic HRs expressed $(His)_6$-NaD1 peptide. Ut: represents non-transgenic HRs.

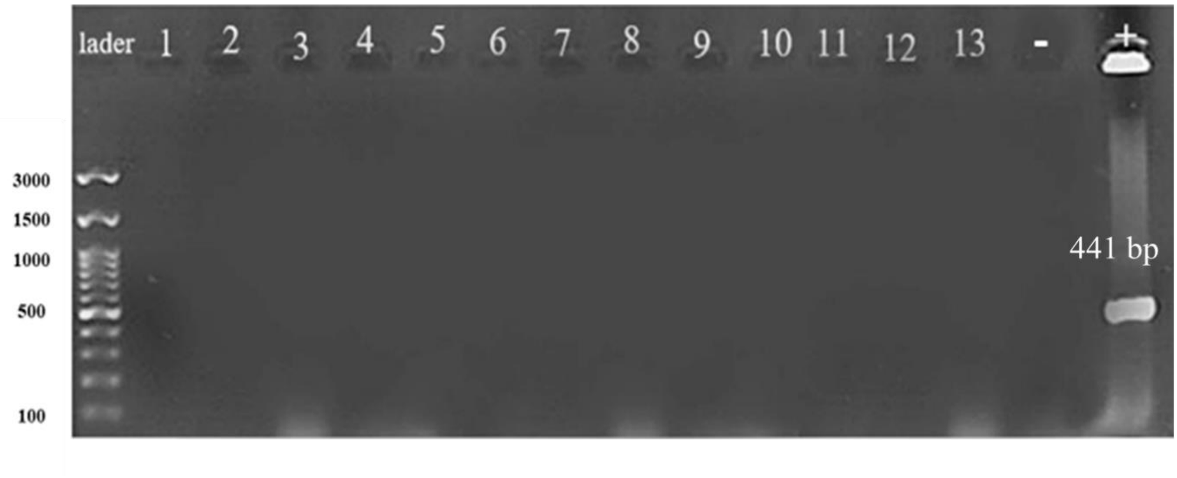

Figure S4. Assessment of bacterial contamination by checking *Vir D1* gene from *Agrobacterium rhizogenes*.

Samples 1 to 4 correspond to transgenic HRs with the *NaD1-(CBD)*$_4$ cassette, samples 5 to 8 to those with the *(CBD)*$_4$*- NaD1* cassette, and samples 9 to 12 to those with the *NaD1* cassette. Sample 13 is the control HR, which lacks the gene fragment. The image also displays the negative control and the amplification band of *Vir D1* at 441 bp, serving as the positive control (*Agrobacterium rhizogenes* PCR). Ladder is 100 bp.

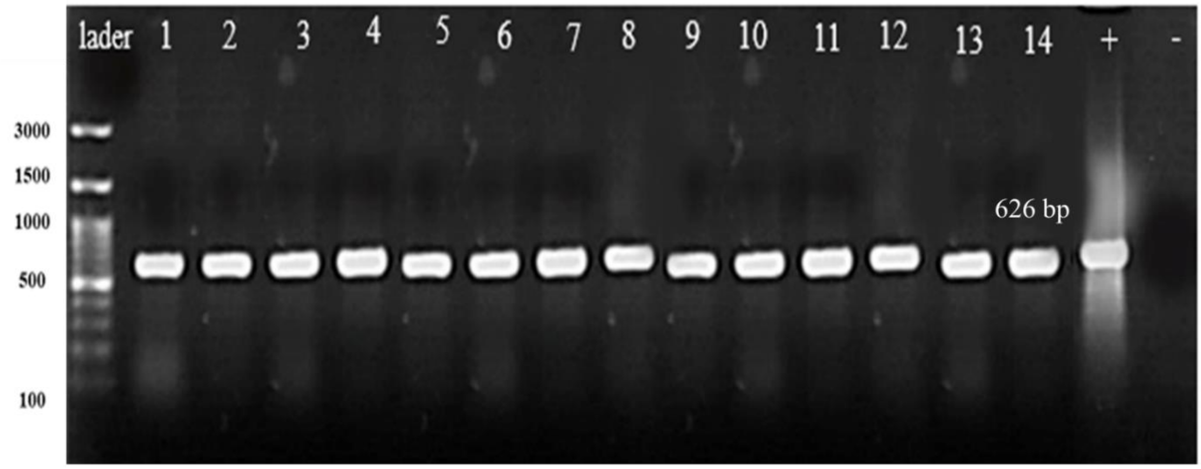

Figure S5. Amplifying of *rol C* gene to confirm the formation of HRs.

Amplifying a 626 bp PCR product of HRs with specific *rol C* gene primers on the 1% agarose gel. Numbers 1 to 5 belong to transgenic HRs containing the *NaD1- (CBD)*$_4$ cassette, numbers 6 to 9 belong to transgenic HRs containing *NaD1-(CBD)*$_4$ cassette, numbers 10 to 13 belong to transgenic HRs containing *NaD1* cassette, number 14 belong to the control HR (lacking the gene fragment). The end of the negative control image and the *rol C* gene bp fragment 626 amplification band are shown as positive control (*Agrobacterium rhizogenes* PCR). The ladder is 100 bp.

S.O.V	DF	Ms			
		2nd day	5th day	7th day	10th day
**Protein extracts**	3	485.9**	1620.8**	755.4**	1131.2**
**Concentrations**	2	817.3**	8.8 n.s	681.5**	470.1**
**Protein extracts *Concentrations**	6	6.3 n.s	27.3**	9.2 n.s	34.6**
**Erorr**	24	8.3	3.1	9.4	8.11
**Total**	35				
**CV**	9.61				

Table S7. Variance analysis of growth inhibition rate of mycelium (*Pyricularia oryzae*) growing on the PDA medium containing concentrations of 20, 50, and 100 µg/ ml NaD1-$(CBD)_4$, $(CBD)_4$-NaD1 and NaD1 and protein extract after 2, 5, 7, and 10 days of culture

.*Significant α=0.05 ** Significant α=0.01 n.s: Non- Significant

S.O.V	DF	Mean squares
**Protein extracts**	4	606.116**
**Time**	2	450.986**
**Protein extracts* Time**	8	79.129**
**Erorr**	30	0.068**
**Total**	44	
**CV**	3.23	

Table S8. Statistical analysis of antifungal activity of the HR protein extracts,

by using the disc diffusion approach against *Pyricularia oryzae*.

*Significant α=0.05 ** Significant α=0.01 n.s: Non- Significant

S.O.V	DF	Mean squares
**Protein treatment**	4	7030**
**Time**	2	2802.222**
**Protein treatment* Time**	8	471.66667**
**Error**	30	66.66667
**Corrected Total**	44	
**CV**	11.62	

Table S9. Variance analysis of the survival percentage of third instar larvae of *Chilo suppressalis* feeding by the recombinant protein extracts after 72 hours.

*Significant α=0.05 ** Significant α=0.01 n.s: Non- Significant

**S.O.V**		**Mean squares**					
	**DF**	**CAT**	**POD**	**APX**	**SOD**	**TRY**	**CTR**
**Protein treatment**	4	0.000011**	112.2**	0.811**	0.000016**	47.88**	28.77**
**Error**	10	0.0000006	0.017	1.72	0.00000016	1.43	0.64
**Total**	14						
**CV**		17.73	14.39	18.6	8.30	12.19	9.88

Table S10 Variance analysis of the activities of CAT, POD, APX, SOD, TRY and CTR enzymes in the third instar larvae of *Chilo suppressalis* feeding by the recombinant protein extracts from tobacco HRs after 72 hours.

*Significant α=0.05 ** Significant α=0.01   n.s: Non- Significant
